# Meta-Analysis of Transcriptomic Data of Dorsolateral Prefrontal Cortex and of Peripheral Blood Mononuclear Cells Identifies Altered Pathways in Schizophrenia

**DOI:** 10.3390/genes11040390

**Published:** 2020-04-03

**Authors:** Maria Cristina Petralia, Rosella Ciurleo, Andrea Saraceno, Manuela Pennisi, Maria Sofia Basile, Paolo Fagone, Placido Bramanti, Ferdinando Nicoletti, Eugenio Cavalli

**Affiliations:** 1 Department of Educational Sciences, University of Catania, 95124 Catania, Italy; m.cristinapetralia@gmail.com; 2 IRCCS Centro Neurolesi Bonino Pulejo, C.da Casazza, 98124 Messina, Italy; rossella.ciurleo@irccsme.it (R.C.); placido.bramanti@irccsme.it (P.B.); 3 Department of Biomedical and Biotechnological Sciences, University of Catania, 95123 Catania, Italy; andresara96@gmail.com (A.S.); manuela.pennisi@unict.it (M.P.); sofiabasile@hotmail.it (M.S.B.); ferdinic@unict.it (F.N.); eugeniocavalli9@hotmail.it (E.C.)

**Keywords:** schizophrenia, prefrontal cortex, transcriptomic meta-analysis, MAP kinases

## Abstract

Schizophrenia (SCZ) is a psychiatric disorder characterized by both positive and negative symptoms, including cognitive dysfunction, decline in motivation, delusion and hallucinations. Antipsychotic agents are currently the standard of care treatment for SCZ. However, only about one-third of SCZ patients respond to antipsychotic medications. In the current study, we have performed a meta-analysis of publicly available whole-genome expression datasets on Brodmann area 46 of the brain dorsolateral prefrontal cortex in order to prioritize potential pathways underlying SCZ pathology. Moreover, we have evaluated whether the differentially expressed genes in SCZ belong to specific subsets of cell types. Finally, a cross-tissue comparison at both the gene and functional level was performed by analyzing the transcriptomic pattern of peripheral blood mononuclear cells of SCZ patients. Our study identified a robust disease-specific set of dysfunctional biological pathways characterizing SCZ patients that could in the future be exploited as potential therapeutic targets.

## 1. Introduction

Schizophrenia (SCZ) is a psychiatric disorder characterized by both positive and negative symptoms, including cognitive dysfunction, decline in motivation, delusion and hallucinations [1]. The worldwide prevalence of SCZ is approximately 1%, with an incidence of about 1.5 per 10,000 people. Typically, the onset is observed between 18 and 25 years for men and between 25 and 35 years for women [2]. The etiology of SCZ is still unknown, with both genetic and environmental factors potentially involved, which may act via functional alteration of the neurotransmitter systems [3].

Despite extensive clinical research, the pathogenic mechanisms underlying SCZ remain elusive. Several cortical and subcortical aberrations have been associated to SCZ, including alterations in morphology, neurotransmitters and physiology [3]. Moreover, polymorphisms in multiple genes have been identified as risk factors for disease development through genome-wide association studies (GWAS) (https://www.ebi.ac.uk/gwas/) [4,5]. The dorsolateral prefrontal cortex (DLPFC) is part of the medial convexity of the frontal lobe and comprises Brodmann areas 9 and 46 and the transitional areas: 9-8, 9-45, 46-10, and 46-45 [6]. The dorsolateral prefrontal cortex (DLPFC) has been described to play a key role in cognitive processes [7]. BA46 is involved in abstract intelligence and metacognition, generating higher-order concepts (i.e., hypothesis) from lower-order concepts, that originate from the BAs enabling social (BA9), material (BA47), and temporal (BA10) cognition (reviewed in [8]). The DLPFC has been shown to be affected in SCZ [9,10,11,12,13], as it plays a key role in verbal and working memory, which are frequently dampened in SCZ [14,15]. Accordingly, several changes have been described in the DLPFC of SCZ patients, such as variation in cell density, in the number of receptors, in gene expression levels, and in proton magnetic resonance (MR) spectroscopy and functional MR imaging studies [10,11,12,16,17,18].

Several studies have explored the gene expression profiles in post-mortem human brain samples, in order to decipher the molecular mechanisms of SCZ; however, molecular markers of the disease and target-tailored treatment strategies are still lacking. The use of meta-analysis of high-throughput expression studies allows one to improve the power and the reliability of the results. In the present study, we have performed a meta-analysis of publicly available whole-genome expression datasets of brain DLPFC cortex, with the aim to prioritize potential pathways underlying SCZ pathology. To improve consistency among studies, we selected only those generated on a specific brain region, and three datasets on Brodmann area 46 were finally used. A functional enrichment analysis was performed to exclude the potential effects of antipsychotic drug treatment. Moreover, we observed that a significant number of differentially expressed genes in SCZ are associated with the expression profiles of astrocytes and neurons. Finally, the comparison of the transcriptomic features of brain and peripheral blood mononuclear cells revealed almost no overlapping among genes and biological processes.

## 2. Materials and Methods

### 2.1. Dataset Selection and Analysis

The NCBI Gene Expression Omnibus (GEO) database (http://www.ncbi.nlm.nih.gov/geo/) was used to identify microarray datasets comparing the transcriptomic profiles of brain from healthy donors and SCZ patients. The GEO database was manually searched using the MeSH term (Medical Subject Headings) “schizophrenia”. The collected datasets were further selected if they met the following inclusion criteria: (a) whole-genome transcriptomic profiling; (b) tissues collected from the dorsolateral prefrontal cortex (DLPFC, defined as Brodmann areas BA9 and BA46, as the DLPFC is considered to be primarily involved in SCZ dysfunction); (c) consisted of both one cohort of SCZ patients and another cohort of healthy people; and (d) species of origin “Homo sapiens.” Finally, three datasets were included in the meta-analysis—GSE53987, GSE12649 and GSE21138—as they were all generated on the BA46 area. Briefly, the GSE53987 dataset included 19 samples from healthy people and 15 samples from SCZ patients [19]. The GSE12649 dataset included 34 healthy control samples and 35 samples from SCZ patients [20]. The GSE21138 dataset included 29 healthy control samples and 30 samples from SCZ patients [21]. The web-based application ImaGEO was used to perform the meta-analysis (http://bioinfo.genyo.es/imageo/) [22]. ImaGEO performs gene expression meta-analysis of GEO microarray datasets by using functions contained in the MetaDE R package [22].

Since all the SCZ subjects recruited for the above-mentioned datasets were exposed to antipsychotic drugs, the potential effects of drug treatment on rat prefrontal cortex was determined by performing a meta-analysis of the GSE2547 and GSE66277 microarray datasets. Briefly, the GSE2547 dataset included data from the frontal cortex of male Sprague-Dawley rats treated with either olanzapine (2 mg/kg/day, I.P.) or saline for 21 days (n = 4 rats per group) [23]. The GSE66277 included whole-genome expression data from male Sprague-Dawley rats treated with either 0.25 mg/kg/day haloperidol, 5 mg/kg/day risperidone or vehicle (1% acetic acid in water) for 21 days (n = 5 rats per group) [19]. For both datasets, the Affymetrix Rat Genome 230 2.0 Array was used.

Next, we wanted to perform a cross-tissue analysis of transcriptomic differences between healthy people and SCZ patients. To this end, the GEO database was searched for whole-genome transcriptomic studies on peripheral blood mononuclear cells (PBMCs) from SCZ patients, and two datasets were found to be suitable, the GSE18312 and GSE27383 datasets. GSE18312 included data from 13 SCZ patients and 8 healthy controls [24], while GSE27383 included PBMCs samples from 43 SCZ subjects and 29 healthy people [25]. The meta-analysis of the two datasets was performed as indicated in the “Statistical Analysis” section, using the ImaGEO software [22].

### 2.2. Evalution of the Involvement of Different Brain Cell Types in SCZ

In order to elucidate the involvement of the different brain cell types in SCZ pathology, we retrieved consensus brain cell gene signatures for astrocytes, microglia, neurons and oligodendrocytes, from the work by McKenzie and colleagues [26]. For the evaluation of the significance of enrichment of brain cell type specific genes, a Chi-square test was performed. The representation factor was defined as the number of overlapping genes divided by the expected number of overlapping genes drawn from the two groups (http://nemates.org/MA/progs/overlap_stats_prog.html). The GeneMania database [27] was used to construct the network of the genes overlapping between a cell-specific signature and the differentially expressed genes (DEGs) from the meta-analysis. Interaction data were defined as physical interaction, co-expression, predicted, co-localization, pathway, genetic interactions, and shared protein domains. The Cytoscape software [28] was used for the visualization of the network with color-coded nodes based on meta-analysis z-values.

### 2.3. Statistical Analysis

For the meta-analyses, a fixed-effect model of effect size measure was used to integrate gene expression patterns from the datasets. Genes with an adjusted *p*-value <  0.05 and an effect size > 2 were identified as DEGs and selected for further analysis. Functional enrichment, interactome analysis, and gene ontology analysis was conducted using the web-based utility, Metascape [29]. Metascape uses the hypergeometric test and Benjamini–Hochberg *p*-value correction algorithm to identify statistically significant enriched ontology terms. Metascape analysis makes use of databases such as Gene Ontology, KEGG, Reactome and MSigDB. Also, Metascape automatically clusters enriched terms into non-redundant groups, by computing pairwise similarities between any two enriched terms based on a Kappa-test score [29]. Unless otherwise specified, an adjusted (Benjamini–Hochberg corrected) *p*-value (adj. *p*-value) < 0.05 was considered as the threshold for statistical significance.

## 3. Results

### 3.1. Identification of a SCZ Gene Profile in BA46

**Figure 1:**
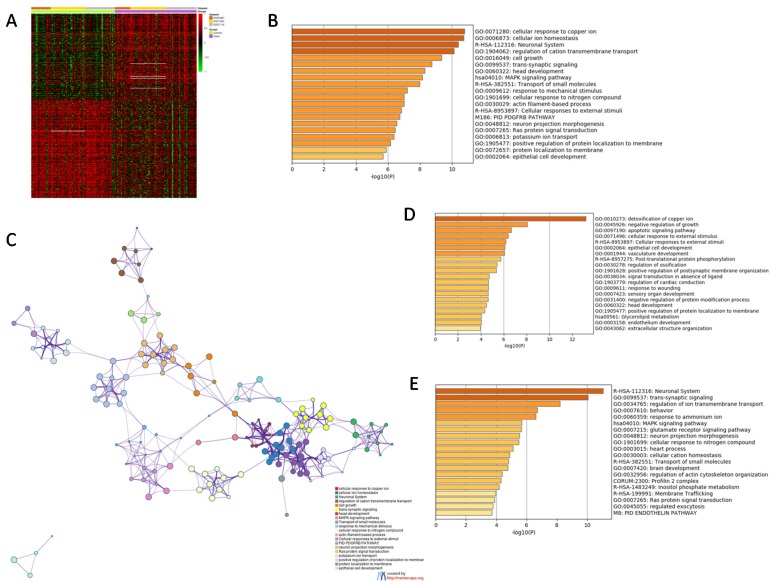
(**A**) Heatmap of the differentially expressed genes (DEGs) identified from the meta-analysis of the GSE53987, GSE12649 and GSE21138 datasets; (**B**) histogram showing the most enriched terms among the DEGs identified in the meta-analysis; (**C**) network showing the interconnection among the most enriched terms among the DEGs identified in the meta-analysis; (**D**) histogram showing the most enriched terms among the upregulated DEGs identified in the meta-analysis; (**E**) histogram showing the most enriched terms among the downregulated DEGs identified in the meta-analysis.

Three GEO datasets were identified for the subsequent evaluation of the transcriptomic differences in BA46 of SCZ patients. A total of 82 samples from healthy controls and 80 SCZ samples were used in the meta-analysis. Overall, 9497 unique genes were included in the meta-analysis. The meta-analysis identified 374 DEGs between the BA46 from healthy and SCZ patients (Figure 1A). Gene ontology analysis revealed that the top enriched terms were: “Cellular response to copper ion” (GO:0071280), “Cellular ion homeostasis” (GO:0006873) and “Neuronal system” (R-HAS-112316) (Figure 1B,C). Among the upregulated DEGs, the top enriched terms were: “Detoxification of copper ion” (GO:0010273), “Negative regulation of growth” (GO:0045926) and “Apoptotic signaling pathway (GO:0097190), followed by “Cellular response to external stimuli” (GO:0071496 and R-HAS-8953897) (Figure 1D). Among the downregulated DEGs, the top enriched terms were: “Neuronal system” (R-HAS-112316), “Trans-synaptic signaling” (GO:0099537), and “Regulation of ion transmembrane transport” (GO:0034765), followed by “Behavior” (GO:0060359) and “MAPK signaling pathway” (hsa04010) (Figure 1E).

**Figure 2:**
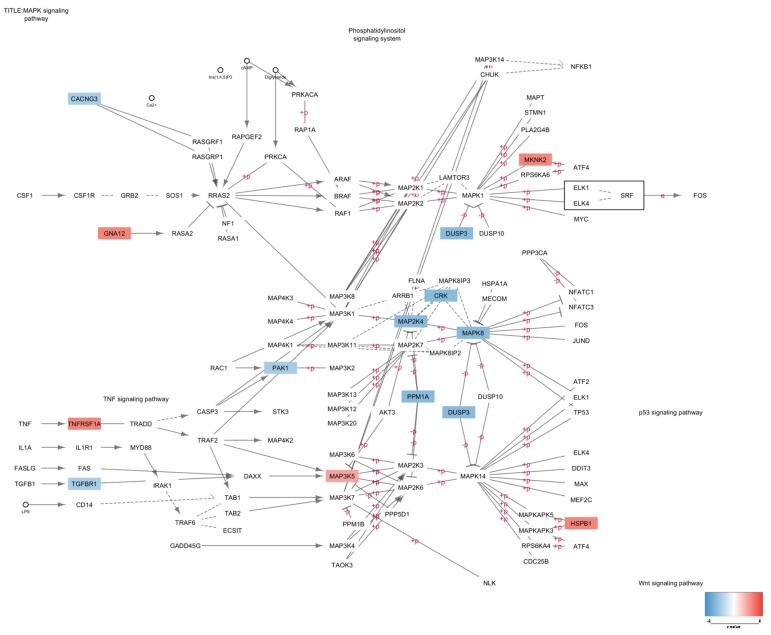
MAPK signaling pathway with nodes color-coded based on the z-value obtained from the meta-analysis of the GSE53987, GSE12649 and GSE21138 datasets.

**Figure 3:**
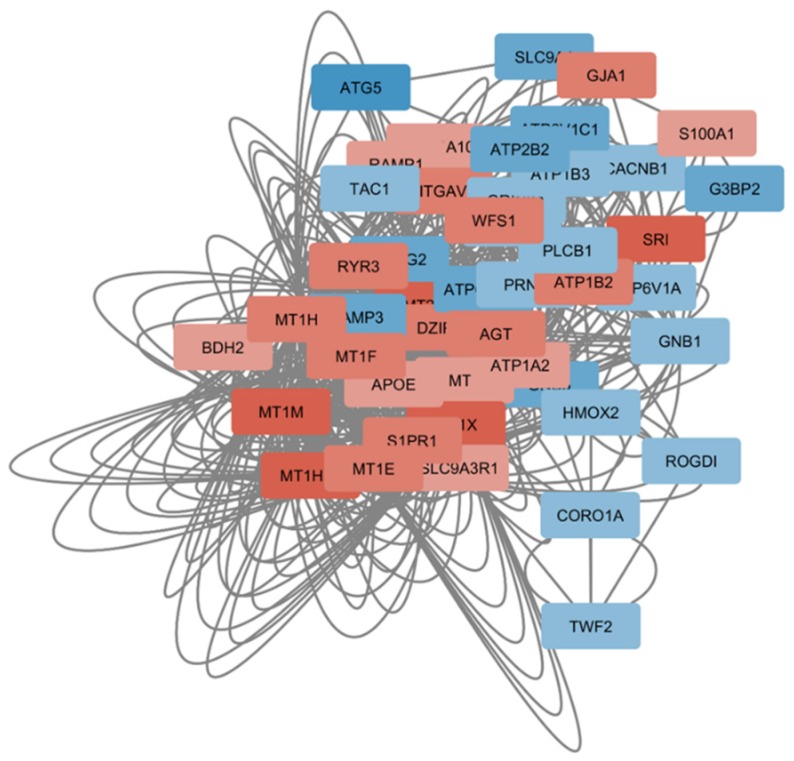
Network constructed using the significantly modulated genes in SCZ and belonging to the “Cellular ion homeostasis” biological process. Nodes are color-coded based on the z-value obtained from the meta-analysis of the GSE53987, GSE12649 and GSE21138 datasets.

Network analysis showed that the two main hub processes were “MAPK signaling pathway” (Figure 2) and “Cellular ion homeostasis” (Figure 3), as they were both connected with four enriched biological processes. In particular, “MAPK signaling pathway” interacted with GO:1901699, M186, R-HAS-8953897 and GO:0009612, while “Cellular ion homeostasis” connected with GO:0071280, GO:1901699, GO:1904062 and GO:0099537 (Figure 1C).

**Figure 4:**
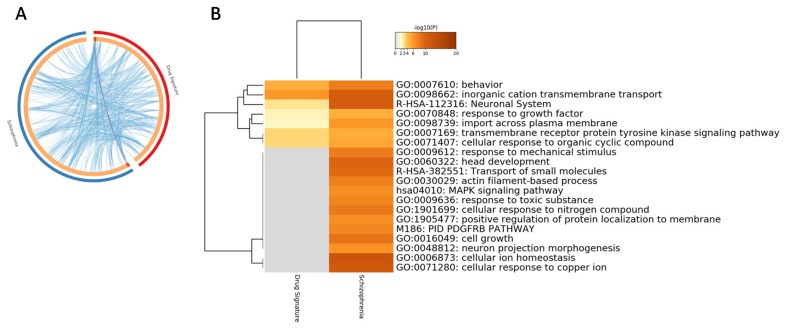
(**A**) Circos plot showing overlapping between the BA46 SCZ differentially expressed genes (DEGs) obtained from the meta-analysis of the GSE53987, GSE12649 and GSE21138 datasets and the antipsychotic drug gene signature, obtained from the meta-analysis of the GSE2547 and GSE66277 microarray datasets. Purple lines link the same genes that are shared by the input lists. Blue lines link the different genes that fall in the same ontology term; (**B**) hierarchical clustering of the top 20 most enriched terms among the DEGs obtained from the meta-analysis of the GSE53987, GSE12649 and GSE21138 datasets and the antipsychotic drug gene signature, obtained from the meta-analysis of the GSE2547 and GSE66277 microarray datasets. The heatmap is colored by the p-values, and grey cells indicate the lack of significant enrichment.

As all the SCZ subjects were under antipsychotic treatment, we aimed at determining whether the enriched pathways identified by the meta-analysis were related to drug exposure rather than to the disease. To this aim, we performed a meta-analysis on the transcriptomic profile of frontal cortex from rats treated with either olanzapine, haloperidol or risperidone. Overall, 15,249 unique genes were included in the meta-analysis. For the current analysis, an adjusted *p*-value < 0.1 was used, as no significant genes were found with a threshold of 0.05. As shown in Figure 3, some of the pathways characterizing the BA46 from SCZ patients were also significantly enriched in the cortex from rats treated with antipsychotic drugs. These enriched terms included: “Behavior” (GO:0060359), “Inorganic cation transmembrane transport (GO:0098662) and “Neuronal system” (R-HAS-112316) (Figure 4). On the other hand, the majority of the pathways significantly altered in SCZ were not affected by drugs in rats (Figure 4).

### 3.2. Cell Type Specific Enrichment

**Figure 5:**
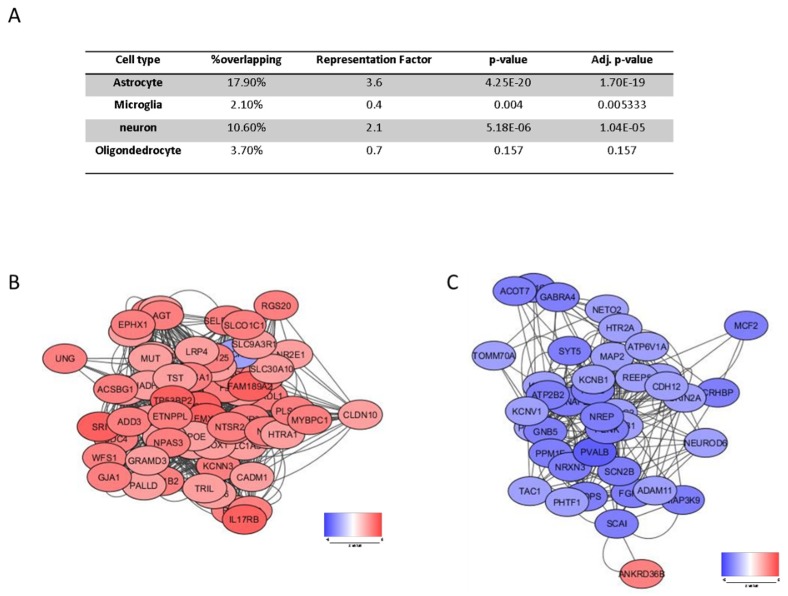
(**A**) Enrichment of brain cell type specific genes among the differentially expressed genes (DEGs) obtained from the meta-analysis of the GSE53987, GSE12649 and GSE21138 datasets; (**B**) network of the astrocyte-specific DEGs obtained from the meta-analysis of the GSE53987, GSE12649 and GSE21138 datasets. The network is color coded by the z value; (**C**) network of the neuron-specific DEGs obtained from the meta-analysis of the GSE53987, GSE12649 and GSE21138 datasets. The network is color coded by the z value. “% overlapping” indicates the percentage of SCZ genes shared with the cell-type specific gene signature; the “representation factor” is the number of overlapping genes divided by the expected number of overlapping genes from the compared groups. A representation factor > 1 indicates more overlap than that expected of two groups, a representation factor < 1 indicates less overlap than expected.

We next wanted to investigate which particular brain cell type is involved in SCZ pathology. Gene enrichment was performed using cell-specific gene signatures previously described [26]. As shown in Figure 5, a significant enrichment for neuronal and astrocyte-specific genes was detected, with 10.6% and 17.9% of overlapping DEGs, respectively (*p* = 5.183e−0.6 and *p* = 4.251e−20 for the neuron- and astrocyte-specific signatures, respectively) (Figure 5A). Interestingly, the majority of the astrocyte-specific genes were upregulated, while most of the neuron-specific genes were downregulated in SCZ (Figure 5B,C). Enrichment analysis of the astrocyte- and neuron-specific DEGs is provided in Table 1 and Table 2.

### 3.3. Perturbed Gene Expression Pattern in SCZ PBMCs and Functional Analysis

Finally, we characterized the SCZ-related blood transcriptomic pattern by performing a meta-analysis of the GSE18312 and GSE27383 datasets. Overall, 15,081 unique genes were included in the meta-analysis (7955 in common with the BA46 meta-analysis) and 176 genes were found to be significantly altered in the PBMCs from SCZ subjects as compared to those from healthy donors. Almost no overlap was found between the BA46-associated and PBMCs-associated DEGs, with the only exception for a consensual upregulation of C11orf68 (Chromosome 11 Open Reading Frame 68). Four other genes were found in common between the brain and blood (i.e., DHRS3, MAML1 SLC9A3R1 and FYN); however, an opposite modulation was observed in the two analyzed tissues, with an upregulation in the brain and a downregulation in the blood. A list of the top 20 most significantly modulated genes in PBMCs of SCZ patients as compared to healthy donors is presented in Table 3.

## 4. Discussion

Dysfunctions in the DLPFC have been described for multiple psychiatric diseases, including SCZ [30,31,32]. The present study shows a profound dysregulation of gene expression in the BA46 of the DLPFC from patients diagnosed with SCZ as compared with control people. Previous high-throughput studies on the prefrontal cortex from SCZ subjects have described modulation of a series of biological pathways associated to synaptic function and signaling pathways [33,34,35,36].

In our study, we observed a significant enrichment in synaptic signaling, as well as downstream effectors of synaptic function, including MAPK signaling and Ras protein transduction. To our knowledge, this is the first meta-analysis to be performed exclusively on studies performed on a specific brain region from SCZ subjects and controls, rather than on studies encompassing different Brodmann areas or brain anatomical structures. The use of whole-genome expression databases has been largely exploited by our group and others [37,38,39,40,41] for the characterization of pathogenic pathways and to identify therapeutic targets for a variety of disorders, such as autoimmune diseases [42,43,44,45,46,47,48,49,50], cancer [44,51,52], and has allowed researchers to characterize pathogenic pathways [53,54,55,56], and potential therapeutic targets [57,58,59,60,61].

A large body of whole-genome profiling studies has been generated in the past few years [62,63,64,65,66]. However, it is difficult to compare the results from the previous studies with our work, since the scopes, aims and methodologies applied are different. Also, a lack of concordance is often observed among these papers. For instance, only four genes were found in common in the TWAS (Transcriptome-Wide Association Studies) analyses presented in the paper from Gusen et al. [62] and Huckins et al. [63], who found 44 and 24 SCZ associated genes, respectively. On the other hand, only 11 genes identified by Hall and colleagues [66] overlapped with genes reported as significant by Huckins and colleagues [63]. As regards the differential expression analysis, while Gandal and collaborators [64] identified 4821 DEGs in the SCZ DLPC (173 in common with our analysis), only 693 were found in Fromer et al. [65] (the complete list of DEGs was not provided, hence no comparison with our data can be made). These discrepancies can largely be attributed to the different inclusion criteria and different modeling approaches used by the authors.

Moreover, the use of bulk DLPC rather than single Brodmann areas does not really allow us to make a comparison between our work and the other studies. Restricting the analysis to a single brain area may significantly minimize the bias of using more heterogeneous samples and facilitates the prioritization of genes and molecular pathways implicated in SCZ pathogenesis. In addition, as technology improves, more cell-specific analyses would be needed for the elucidation of SCZ-related etiological mechanisms and the relative contribution of each cellular type.

Our meta-analysis shows that the significantly upregulated genes in the SCZ have functions in the regulation of cell survival and growth and in response to external stimuli, while the downregulated genes are involved in neuronal homeostasis and intracellular signaling, including the MAPK pathway. The MAPK pathway is known to activate transcription factors involved in cell proliferation and survival, learning and memory. This pathway integrates extracellular stimuli via the phosphorylation of c-Jun N-terminal kinase (JNK), extracellular signal-regulated kinase (ERK), and p38, among others [67,68,69]. Alterations to the MAPK signaling may therefore affect biological processes critical for the maintenance of neuroplasticity [70,71,72]. This finding is in accordance with previous observations on the association between MAPK3 [62], MAP2K7 [73] and MAPK4 [74] and SCZ. Moreover, abnormal activity of MAPK signaling has been observed in frontal cortical areas from postmortem brains of SCZ subjects [75] and, in a focus quantitative proteomic study, the GNA13-ERK1-eIF4G2 signaling was detected to be downregulated [76]. It should, however, be pointed out that the downregulation of the MAPK-associated signaling may be specific to certain brain areas, and different patterns of modulation can instead be observed in other regions. Indeed, a significant increase in ERK2, c-fos and c-jun protein and mRNA levels has been reported in the thalamus of patients with schizophrenia relative to controls [67]. It is worth noting that the alteration of the MAPK signaling pathway in SCZ may partly explain the negative correlation between SCZ and rheumatoid arthritis (RA), as a large body of data points to the pathogenetic role of MAPK signaling in RA [77,78,79,80]. Chen et al. reported a lower incidence of RA in patients with SCZ after adjustment for demographics and comorbidities [81]. Moreover, the presence of RA predicted a reduced, although not significant, incidence rate for SCZ [81]. This is also in line with the observation of a negative correlation between gene expression patterns in SCZ and RA [82], as well as with the small but significant negative SNP-genetic correlation between these diseases [83]. It should however be noted that we failed to observe a reduction in the expression of most MAP kinases (with the only exception being MAPK7) in the peripheral blood of SCZ patients. This seems to be in contrast with the possibility that downregulated MAPK expression and function may protect SCZ patients from RA, as it seems logical that a reduced expression of MAPK in peripheral blood or joints would be needed to protect from RA development. Vice versa, it seems doubtful that selective impaired MAPK activity in the CNS may play an anti-arthritogenic role. Hence, while we provide evidence for a downregulated expression of MAPK in brain of SCZ patients, the eventual role that divergent MAPK expression in SCZ and RA may play in the negative association of the two diseases remain to be established. It seems, on the other hand, of some interest that SCZ patients seem to have a reduced incidence of gliomas. Previous epidemiological studies showed that persons with schizophrenia may be less likely to suffer from gliomas [84,85]. This has recently been hypothesized to depend on the fact that glioma tumorigenesis and schizophrenia may share similar mechanisms, and the molecular defects of certain molecules, such as DISC1 (Disrupted in schizophrenia 1), P53 (also known as TP53, Tumor Protein 53), BDNF (Brain-derived neurotrophic factor) and CXCR4 (C-X-C chemokine receptor type 4) involved in SCZ pathogenesis might play opposite roles in glioma development. It may be possible and worth further investigation to evaluate whether reduced CNS expression of MAPK may represent an additional protective factor. From the translational point of view, our analysis suggests that compounds able to upregulate MAPK expression may be of interest for preclinical pharmacological studies.

Our study has also detected a significant alteration in the regulation of ion homeostasis in the brain from SCZ patients. This is in line with reports showing that an aberrant expression of several metallothioneins can be found in the DLPC of psychosis patients [86] and that the concentration of zinc is altered in several of disorders of the central nervous system, including SCZ (reviewed in [87]). Also, we observed a significant modulation in the expression levels of several ATPases in SCZ brains, supporting previous preclinical and clinical data on the role of neuronal ATP turnover in neuropsychiatric diseases [88,89,90,91,92,93].

The meta-analysis of the PBMC-related transcriptional profile yielded less significantly dysregulated genes as compared with the brain-related analysis. This observation may suggest that epigenetic-driven changes occurring in the brain may exert a more prominent etiopathogenetic trigger in the development/maintenance of SCZ than genetic influence, which would had been reflected in the peripheral tissues. In accordance with this hypothesis is the observation that no cross-tissue overlap was observed at either the level of dysregulated gene lists or at the functional level. Hence, we may speculate that genetic elements may play a relatively minor role in modeling the SCZ-associated transcriptomic features.

Since all the samples used in our analysis were from SCZ patients under pharmacological treatment, we integrated data from two microarray datasets generated on the frontal cortex of rats chronically treated with either haloperidol, risperidone or olanzapine. A few of the genes differentially modulated upon medication were in common to the SCZ gene signature and, more specifically, were related to pathways associated with neuronal function and trans-membrane transport. These data suggest that many of the processes enriched in SCZ are not linked to medication but may independently underlie SCZ pathology. There are, however, some caveats to take into consideration. First, the drug signature was generated from rats treated with a limited number of antipsychotics, i.e., haloperidol, risperidone and olanzapine. Moreover, the effect of the concomitant administration of multiple drugs, which is common in psychiatric patients, cannot be evaluated in our present analysis and, therefore, their eventual combined effects on transcriptomic profiles cannot be excluded. Second, the drugs were administered to healthy rats, without any neurological disorder resembling SCZ. Therefore, many potential pathways affected by the medication in patients could have been missed. Third, the frontal cortex from the rodents does not accurately match the human DLPFC. Fourth, we cannot determine whether the pathways altered upon drug treatment represent the therapeutic response to the medication. Hence, a cause-effect relationship cannot be established.

Our data suggest that two brain cell types are affected in SCZ, i.e., astrocytes and neurons. Interestingly, we observed the upregulation of most of the astrocyte-specific genes, and a downregulation of the majority of the neuron-specific genes. Whether these changes are associated to a variation in cell type-specific abundance has yet to be deciphered. However, our study supports an emerging agreement on the role of cortical interneurons and astrocytes in SCZ [94]. In particular, Toker and colleagues [94] reported an increase in astrocyte and a decrease in fast-spiking parvalbumin-positive interneuron markers in the neocortex of both SCZ and bipolar patients. Whether the modulation of these markers correlates with altered cell type proportions rather than with changes in cellular phenotypes is yet to be determined. Of note, several studies have not detected SCZ-associated changes in the astrocyte-marker GFAP (Glial Fibrillary Acidic Protein), suggesting that astrocyte proportions may not be changed in SCZ, while other studies showed either decreased or increased expression levels of GFAP (reviewed in [95]). On the other hand, other astrocytic markers have been investigated in postmortem brain samples from SCZ patients, showing an increase in the expression of apolipoprotein 1 and adenosine A2A receptor, as well as augmented levels of aldehyde dehydrogenase vimentin, excitatory amino-acid transporter and phosphate-activated glutaminase [95].

The abovementioned data strongly support the need to focus future studies on cell–specific changes rather than bulk tissue data. Also, it would be advisable to perform additional evaluation of the differential gene expression between the right and left hemispheres of the prefrontal cortex in order to better characterize the mechanisms leading to SCZ.

## 5. Conclusions

Overall, with the present study, we are providing a unified evaluation of previous studies on the gene signatures characterizing the BA46 area of patients with SCZ. However, whether these changes are drivers of the disease or consequences of genetic/epigenetic factors remains to be determined. Following the identification of the pathogenetic pathways characterizing SCZ, the use of in silico pharmacology and drug discovery approaches will help to design tailored pharmacological strategies that may regulate these pathways.

## Figures and Tables

**Table 1:** Top 10 enriched terms among the astrocyte-specific DEGs.

GO	Description	Count	%	Log10 (*p*-Value)	Log10 (adj. *p*-Value)
GO:0010959	regulation of metal ion transport	10	15.15	−6.95	−2.79
GO:0044282	small molecule catabolic process	10	15.15	−6.44	−2.75
GO:0009612	response to mechanical stimulus	7	10.61	−5.77	−2.62
GO:0051047	positive regulation of secretion	9	13.64	−5.61	−2.54
GO:0019932	second-messenger-mediated signaling	9	13.64	−5.52	−2.51
R-HSA-382551	Transport of small molecules	11	16.67	−5.39	−2.41
GO:0007423	sensory organ development	9	13.64	−4.76	−2.03
GO:0034368	protein-lipid complex remodeling	3	4.55	−4.17	−1.65
GO:0050954	sensory perception of mechanical stimulus	5	7.58	−4.05	−1.58
GO:0009636	response to toxic substance	8	12.12	−4.05	−1.58

**Table 2:** Top 10 enriched terms among the neuron-specific DEGs.

GO	Description	Count	%	Log10 (*p*-Value)	Log10 (adj. *p*-Value)
GO:0007268	chemical synaptic transmission	14	35.00	−11.47	−7.64
R-HSA-112316	Neuronal System	10	25.00	−8.99	−5.37
GO:0098662	inorganic cation transmembrane transport	12	30.00	−8.72	−5.18
GO:0043269	regulation of ion transport	11	27.50	−7.91	−4.50
GO:0007610	behavior	10	25.00	−7.47	−4.11
GO:0072347	response to anesthetic	4	10.00	−5.07	−2.22
R-HSA-422356	Regulation of insulin secretion	4	10.00	−5.07	−2.22
GO:0035637	multicellular organismal signaling	5	12.50	−4.66	−1.88
hsa04726	Serotonergic synapse	4	10.00	−4.44	−1.70
GO:0001508	action potential	4	10.00	−4.16	−1.49

**Table 3:** Top 20 most significantly modulated genes in PBMCs of schizophrenia patients as compared to healthy donors, as determined from the meta-analysis of GSE18312 and GSE27383 datasets.

ID	Adj. *p*-Value	*p*-Value	z-Val	Gene_Name
GPR15	0.00754	1.00E−06	4.891597	G protein-coupled receptor 15
ICAM2	0.00754	5.99E−07	−4.99165	intercellular adhesion molecule 2
KLHL21	0.013174	4.37E−06	−4.59301	kelch like family member 21
RANGAP1	0.013174	3.01E−06	−4.67032	Ran GTPase activating protein 1
UBA3	0.013174	4.11E−06	4.605957	ubiquitin like modifier activating enzyme 3
MAPK7	0.013234	6.14E−06	−4.52136	mitogen-activated protein kinase 7
SCAP	0.013234	5.60E−06	−4.54101	SREBF chaperone
EAF2	0.018421	1.10E−05	4.396598	ELL associated factor 2
MAML1	0.018421	1.02E−05	−4.41356	mastermind like transcriptional coactivator 1
CCDC107	0.019182	1.50E−05	−4.3293	coiled-coil domain containing 107
FAM193A	0.019182	1.94E−05	−4.27156	family with sequence similarity 193 member A
KIF21B	0.019182	1.53E−05	−4.32392	kinesin family member 21B
RNF44	0.019182	1.93E−05	−4.27328	ring finger protein 44
SELPLG	0.019182	1.58E−05	−4.31701	selectin P ligand
TCL1B	0.019182	1.80E−05	4.288751	T cell leukemia/lymphoma 1B
ZNF76	0.019182	2.04E−05	−4.26095	zinc finger protein 76
ABHD15	0.019623	2.50E−05	−4.21456	abhydrolase domain containing 15
MEF2D	0.019623	2.67E−05	−4.19974	myocyte enhancer factor 2D
SEC24C	0.019623	2.73E−05	−4.19463	SEC24 homolog C, COPII coat complex component
TMEM123	0.019623	2.33E−05	4.230499	transmembrane protein 123
